# Enzyme-Responsive Materials as Carriers for Improving Photodynamic Therapy

**DOI:** 10.3389/fchem.2021.763057

**Published:** 2021-11-02

**Authors:** Houhe Liu, Fanwen Yang, Wenjie Chen, Teng Gong, Yi Zhou, Xiaoyan Dai, Wingnang Leung, Chuanshan Xu

**Affiliations:** ^1^ Key Laboratory of Molecular Target and Clinical Pharmacology, State Key Laboratory of Respiratory Disease, School of Pharmaceutical Science and Fifth Affiliated Hospital, Guangzhou Medical University, Guangzhou, China; ^2^ Department of Biomedical Engineering, School of Basic Medical Sciences, Guangzhou Medical University, Guangzhou, China; ^3^ School of Nursing, Tung Wah College, Hung Hom, Hong Kong, SAR China

**Keywords:** photodynamic therapy, photosensitizer, smart materials, enzyme-responsive materials, carrier

## Abstract

Photodynamic therapy (PDT) is a mini-invasive therapy on malignancies via reactive oxygen species (ROS) induced by photosenitizer (PS) upon light irradiation. However, poor target of PS to tumor limits the clinical application of PDT. Compared with normal tissues, tumor tissues have a unique enzymatic environment. The unique enzymatic environment in tumor tissues has been widely used as a target for developing smart materials to improve the targetability of drugs to tumor. Enzyme-responsive materials (ERM) as a smart material can respond to the enzymes in tumor tissues to specifically deliver drugs. In PDT, ERM was designed to react with the enzymes highly expressed in tumor tissues to deliver PS in the target site to prevent therapeutic effects and avoid its side-effects. In the present paper, we will review the application of ERM in PDT and discuss the challenges of ERM as carriers to deliver PS for further boosting the development of PDT in the management of malignancies.

## Introduction

**FIGURE 1:**
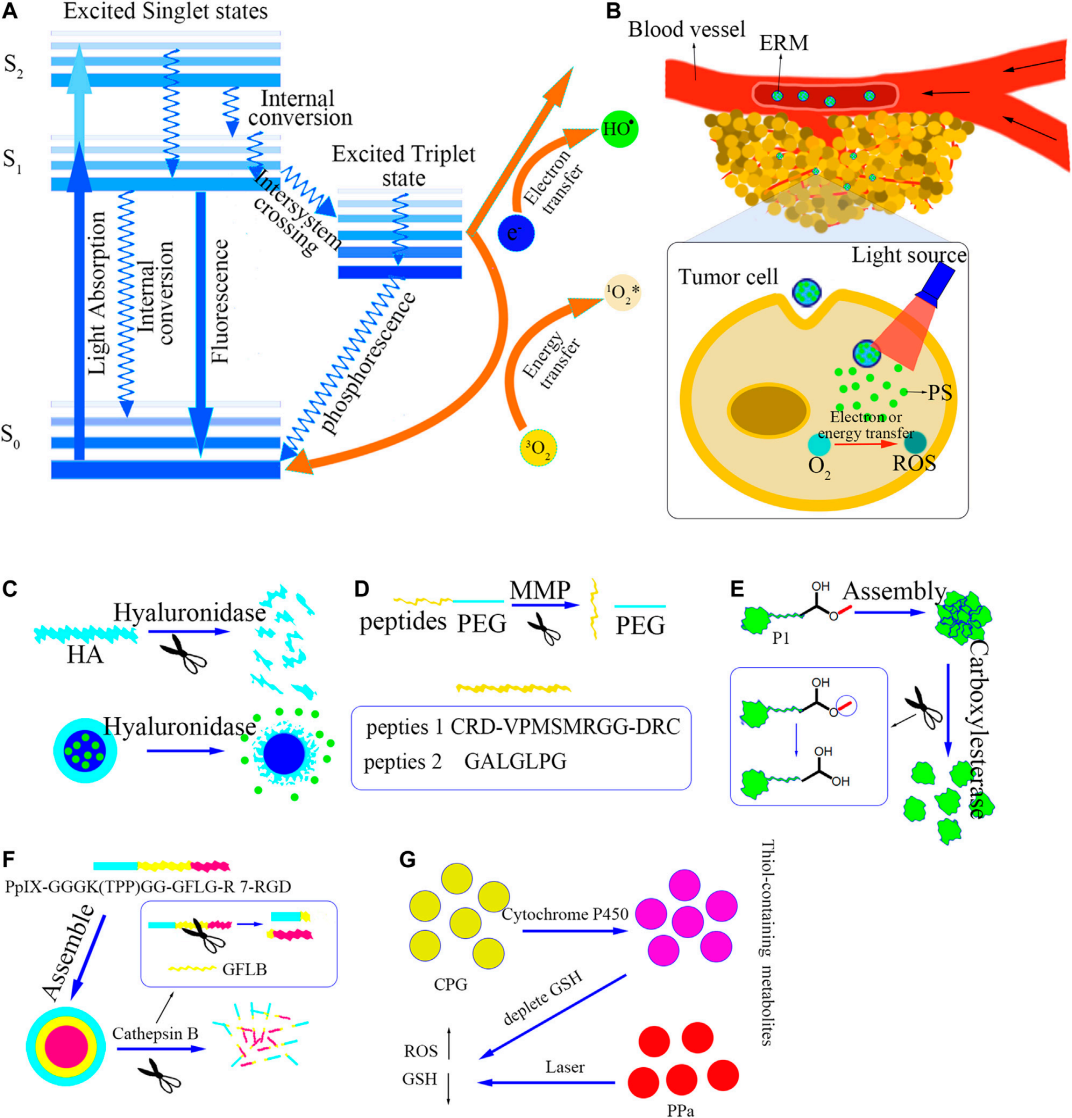
**(A)** The principle of ROS generated by photosensitizer excitation. **(B)** The process of ERM delivering PS. **(C-G)** The response principles of the five ERMs, followed by hyaluronidase-responsive, Matrix metalloproteinase-responsive, Carboxylesterase-responsive, Cathepsin B-responsive and Cytochrome P450-responsive.

Cancer is one of the most threatening diseases to humans (Zhang et al., 2019). Chemotherapy is one of the main treatments in the management of malignancies, but chemotherapy induced side effects have severely affected the therapeutic outcome due to non-target accumulation and drug resistance (Xing et al., 2019; Jiang et al., 2020). In particular, the systemic toxicity of conventional chemical drugs significantly limited the development of chemotherapy (Li et al., 2021). Photodynamic therapy (PDT) is being paid wide attention as a non-invasive, low-toxicity, and high-efficient treatment approach (Chi Zhang et al., 2019; Izumoto et al., 2020). As early as thousands of years ago, the ancients used herbs and sunlight together, but they did not cause concern (Bown, 2013; Zhao and He, 2014). Until the beginning of the twentieth century, scientists discovered that exposing paramecium that swallowed photosensitizer (PS) to visible light caused death (Dolmans et al., 2003; Diogo et al., 2015; Mansoori et al., 2019). PDT is based on the production of a large amount of ROS to kill tumor cells through light-activated PS (Fan et al., 2016; Wu et al., 2019; Zhou et al., 2020). As Figure 1A shown, while the PS is exposed to light with a specific wavelength, the outermost electron in the molecular orbital transitions from the ground state S_0_ to the excited singlet state (Ackroyd et al., 2001). Then, intersystem crossing or spin reversal occurs, and the molecule transforms into an excited triplet state with a longer lifetime (Mansoori et al., 2019). These two unstable states of PS lose energy through the emission of fluorescence or phosphorescence and the internal conversion into heat (Muniyandi et al., 2020). The PS in the excited triplet state can produce photochemical reactions, including type I reaction or type II reaction. The excited PS reacts with the molecules (including oxygen) in the surrounding environment through the electron transfer process, resulting in free radicals production (Schalch et al., 2019). These free radicals produce toxic effects by destroying biological macromolecules in tumor cells. It is termed as the type I reaction (Debele et al., 2015). On the other hand, in the type II reaction the activated PS generates excited singlet oxygen (^1^O_2_) through the direct energy transfer from the triplet state PS to the triplet oxygen (Dang et al., 2017). Therefore, the accumulation of PS in tumor cells is the premise of PDT, and light and molecular oxygen are indispensable conditions for PDT (Pucelik et al., 2020).

PDT has recently shown promise in the treatment malignancies because of its high efficiency and safety. However, the hydrophobicity and poor targeting of PS need to be urgently addressed (Sivasubramanian et al., 2019). There are two main ways to solve them. Structural modification is a commonly used method to develop a new type of small molecule PS with excellent properties. At present, the use of responsive materials as delivery carrier provides noval strategy for addressing the hydrophobicity and poor targeting of PS (Bowman and Leong, 2006; Sheng et al., 2016; Zhang et al., 2018; Lin et al., 2021). Especially, tumor microenvironment-responsive material (TMRM) as an interesting delivery carrier is attracting extensive attention (Liu et al., 2020). TMRM can respond to the unique physiological environment of tumors, thereby selectively releasing PS and enhancing PDT (Torchilin, 2014). TMRMs mainly include hypoxia-responsive materials, pH-responsive materials, redox-responsive materials and enzyme-responsive materials (ERMs), etc (Wells et al., 2019).

Enzymes are essential for biological activities, and some uncontrolled and highly expressed enzymes in tumors are potential targets for stimulus response (Mura et al., 2013; Du and Tung, 2020). A large number of ERMs have been designed for use in drug delivery systems (Li et al., 2020; Mi, 2020). In PDT, ERM can also be used to deliver photosensitizers selectively to the tumor site, subsequently promoting the accumulation of PS in the tumor tissues and enhancing PDT efficiency through the enzymatic response to cause the transformation of intratumoral substances. In the present paper, we will review the application of ERM in PDT and discuss the challenges of ERM as carriers to deliver PS for further boosting the development of PDT in the management of malignancies.

### Enzyme-Responsive Materials

ERM is a smart material that can respond to different enzymatic environments. ERMs have been widely used as a carrier for delivering PS to tumor tissues. As Figure 1B shown, after the PS-loaded ERM enters the tumor tissues, the PS can be specially released from the ERM by responding to the unique enzymes in the tumor tissues, and then the use of light irradiation activates the PS to produce ROS to kill the tumor cells. Recently, the reported ERMs used for PS delivery mainly include hyaluronidase-responsive material, matrix metalloproteinase-responsive material, carboxylesterase-responsive material, cathepsin B-responsive material, cytochrome P450-responsive material, etc. Their detailed information of the response sites and response principles is shown in Table 1.

**TABLE 1:** Summary of enzyme-response materials for PDT.

Types of enzyme response materials	Materials	Photosensitizer	Responsive site	Principle of response	Application	Reference
Hyaluronidase-responsive	MTM	TiO_2_	hyaluronic acid	Hyaluronidase causes HA degradation	PDT for B16-F10 cells/PDT for B16-F10 tumors	Zhou et al. (2020)
	HA-TPZ and IR@GMON	IR820	hyaluronic acid	Hyaluronidase causes HA degradation	PDT for 4T1, Lo2 and L929 cells/PDT for 4T1 tumors	Cheng et al. (2021)
Matrix metalloproteinase-responsive	Hydrogel	OPVBT	CRD-VPMSMRGG-DRC	MMP cleavable the sequence of CRD-VPMSMRGG-DRC	PDT for 293T, MCF-7 and MDA-MB-231 cells	Li et al. (2017)
	EAPV	PPa	GALGLPG	MMP-2 cleavable the sequence of GALGLPG	PDT for CT26 cells/PDT for CT26 tumors	Gao et al. (2019)
Carboxylesterase-responsive	FHP	PMI	Carboxylic acid ester bond	Carboxylic acid ester bond selectively hydrolyzed by carboxylesterase	PDT for Hepg2, 293T and Bel-7404 cells/PDT for H22 tumors	Cai et al. (2020)
Cathepsin B-responsive	PpIX-GGGK (TPP)GG-GFLG-R 7 -RGD	PpIX	GFLG	Cathepsin B-cleavable the linker of GFLG.	PDT for COS 7 and HeLa cells	Cheng et al. (2020)
Cytochrome P450-responsive	CPG/PPa NPs	PPa	CPG	Cytochrome P450 (CYP2C19) enzyme mediates the metabolism of CPG to form thiol-containing metabolites	PDT for 4T1 and RM-1 cells/PDT for 4T1 tumors	(Qiu Wang et al., 2020)

### Hyaluronidase-Responsive Material

Hyaluronidase is an enzyme that can decompose high-molecular hyaluronic acid into small-molecule hyaluronic acid (Lv et al., 2015). Growing evidence shows that hyaluronidase is a potential cancer biomarker and therapeutic target (McAtee et al., 2014). Hyaluronic acid (HA) is a water-soluble mucopolysaccharide, which is a ligand specifically binding to CD44 overexpressed in cancer cells (Choi et al., 2011; Yu et al., 2013; Dosio et al., 2016; Liu et al., 2020). Therefore, it is a reasonable choice to use materials containing hyaluronic acid to target tumor cells and/or to release photosensitizers in response to hyaluronidase in tumor tissues. Zhou (Zhou et al., 2020) constructed a mesoporous TiO_2_ doped with MnO_2_ (MTM) through an improved sol-gel method to develop co-doped photosensitizer TiO_2_ for enhancing PDT in the treatment of melanoma. MTM was also loaded with the antioxidant scavenger Au 25 Sv 9 and then modified by hyaluronic acid on the surface of MTM to avoid accidental release of Au 25 Sv 9. As Figure 1C shown, after the HA-modified MTM entering the tumor tissues, hyaluronidase decomposes hyaluronic acid to release Au 25 Sv 9 to remove thioredoxin reductase in tumor cells, MnO_2_ catalyzes H_2_O_2_ to produce oxygen to improve hypoxia environment, which promoted the killing effect of photodynamic action through light-excited TiO_2_. The authors found that the composite materials could improve hypoxia in tumor tissues and enhance PDT efficacy.

The use of hyaluronic acid as a sealing material of mesoporous silica can not only target CD44 receptors overexpressed in tumor cells, but also control the release of photosensitizers through enzyme response. Cheng (Cheng et al., 2021) reported a new type of nanohybrid based on GSH-responsive mesoporous organic silica nanoparticles (GMON), which could exert significant anti-tumor activity under near-infrared light irradiation. The nanosystem was composed of GSH-responsive mesoporous silica, photosensitizer IR820, hypoxia-responsive prodrug tirapazamine (TPZ) and hyaluronic acid. After administration, the nanosystem was endocytosed into tumor cells due to HA targeting CD44 receptor. The GMONs in tumor cells were degraded by the action of GSH and hyaluronidase, releasing TPZ and photosensitizer. After laser irradiation IR820 consumed oxygen to produce ROS, which further enhances the toxic effect of TPZ to tumor cells. HA has dual ability to target CD44 overexpressed in cancer cells and response to hyaluronidase overexpressed in cancer cells, which endows HA-modified materails double functions with targeted release and delivery of PS. At present, electrostatic absorption is a main method used for the modification of HA on the surface of nanomaterials. The electrostatic force is very weak and the modified nanomaterials are easily affected by the surrounding envirnoments. After entering the systemic circulation, HA on the surface of the nanomaterials is easy to drop from the materials and cause accidental release of PS. Therefore, if HA is modified on the nanomaterials through chemical bonds, the physicochemical properties and biological activity will be much improved.

### Matrix Metalloproteinase-Responsive Material

Matrix metalloproteinases (MMP) are overexpressed in a variety of tumors, and are closely related to tumor growth, invasion and metastasis (Shinagawa et al., 1994; Coussens et al., 2002; Natarajan et al., 2006; Roy et al., 2008). A large number of anti-cancer strategies related to MMP have been proposed, especially MMP sensitive or responsive substances have been widely used to deliver drugs and reduce the side effects of drugs (Winer et al., 2018). In PDT, MMP is also used as an excellent stimulus source to targetedly deliver photosensitizers. As an important subcellular organelle, mitochondria are related to cell growth, differentiation and apoptosis. The development of photosensitizers with mitochondrial targeting is an effective way to enhance photodynamic therapy. (Li et al., 2017) synthesized a cationic oligomer (p-phenylene vinylene-copolymerized benzothiazole) (OPVBT) that could accumulate in mitochondria via electrostatic interactions. OPVBT could generate a large amount of ROS upon light irradiation and exhibited significant photodynamic activity. In their studies MMP-sensitive PEG peptide (CRD-VPMSMRGG-DRC) hydrogel was used to deliver OPVBT for improving tumor selectivity and reducing side effects. Due to the high level of MMP in tumor cells, hydrogels containing MMP-cleavable (Figure 1D) substrates could gradually be degraded, locally releasing OPVBT in tumor tissues. Their results showed that the hydrogel had no toxicity to normal cells, but it could be effectively decomposed after entering tumor tissues, to release the photosensitizer, showing high selectivity and low toxicity.

MMP-2 is one of MMPs, which are overexpressed in a variety of tumor cells (Wang et al., 2019; Stephens et al., 2020). (Gao et al., 2019) designed MMP-2 responsive exfoliable prodrug vesicles (EAPV) containing pegylated photosensitizer and NLG919 prodrug (NPC). PEG was modified on the surface of the vesicle through the MMP-responsive linker—GALGLPG (A short peptide), and when the vesicles reached tumor site, the GALGLPG peptide (Figure 1D) was cleaved by MMP-2 and then removed PEG to increase the endocytosis of the vesicles. After entering tumor cells, the loaded drug NLG-919 was released in response to GSH to inactivate indoleamine 2,3-dioxygenase 1, thereby overcoming the adaptive immune resistance caused by PDT. The results demonstrated that the EAPV nanoviscles exhibited uniform size distribution, good serum stability, and dual responsiveness to MMP-2 and GSH. EAPV could also penetrate deeply into tumor tissues and be easily absorbed by tumor cells. The *in-vitro* and *in-vivo* experiments confirmed that EAPV could significantly produce ROS, induce tumor cell ICD, and ultimately enhance the immune recognition of dendritic cells to tumor cells.

### Carboxylesterase-Responsive Material

Carboxylesterase (CE) is a member of α/β hydrolase folding protein, which is mainly located in the endoplasmic reticulum of various mammalian cells (Barthel et al., 2009; Löser et al., 2013). CE participates in the hydrolysis of many xenobiotics and endogenous ester or amide compounds, and plays a key role in the metabolism and biotransform of some chemotherapeutics (Ke et al., 2020). Studies have shown that CEs are highly expressed in a variety of human tumor cells, so CE is also used as a target stimulus source to deliver drugs (Sanghani et al., 2003). Cai (Cai et al., 2020) modified the tetrachloroethylene monoimine core by β-alanine methyl ester, synthesized the CE-responsive precursor P1 as the substrate of CE and the electron donor of the system, and galactose as the tumor targeting ligand and hydrophilic group. The synthesized near-infrared (NIR)-activated amphiphile P1 and folate-modified human serum albumin (HSA) could assemble into nanoclusters (FHP) with a size of about 100 nm. As Figure 1E shown, when the methyl carboxylate of P1 was selectively hydrolyzed by CE at the tumor site to remove the methyl group, FHP decreased from ∼100 to ∼10 nm, which was conducive to the penetration of FHP in the deep tissues of the tumor. At the same time, the enzyme-triggered decomposition of FHP enhanced near-infrared fluorescence (NIRF) and promoted the generation of singlet oxygen (^1^O_2_), thereby improving imaging-guided PDT.

### Cathepsin B-Responsive Material

The expression of cathepsin B is highly upregulated in malignant tumors and precancerous lesions, so cathepsin B can be used as a stimulus response target (Melancon and Li, 2011; ShiJie; Wang et al., 2020). Given that high levels of Cathepsin in tumor tissues can specifically cleave a tetra-peptide linker GFLG (Özel et al., 2015), Cheng (Cheng et al., 2020) constructed a tumor cell and mitochondrial dual-targeting nano-platform based on PpIX-GGGK (TPP)GG-GFLG-R7-RGD for the co-delivery of DOX and protoporphyrin IX (PpIX). The self-assembled nano-platform consisted of a hydrophobic PpIX-peptide conjugate (PpIX-GGGK (TPP)GG), a hydrophilic bioactive peptide with tumor-targeting RGD, cell membrane-penetrating R8 and cathepsin B. The cleaved linker GFLG is composed of self-assembled nanomicelles with a core-shell structure using hydrophilic-hydrophobic interactions, and DOX is loaded into the core through hydrophobic force and π-π interactions to form DOX@pPAC. The constructed nanosystem (DOX@pPAC) enhanced the uptake of PpIX and DOX in tumor cells through endocytosis mediated by integrin receptors. Later, the up-regulated lysosomal protease cathepsin B triggered the hydrolysis of the GFLG bond, followed by the dissociation of the DOX@ pPAC nanomicelles (Figure 1F). Then, the TPP cation of the PpIX-peptide conjugate greatly promoted the accumulation of PpIX in mitochondria, and significantly enhanced the PDT effect of PpIX on tumor cells. In this article, the researchers cleverly designed a multifunctional amphiphilic macromolecule that could self-assemble, integrating PDT, DOX, tumor targeting, mitochondrial targeting and enzyme response. The successful combination of chemotherapy and PDT amplifies the efficacy of chemotherapy and photodynamic therapy and accelerates the death of tumor cells.

### Cytochrome P450-Responsive Material

Cytochrome P450 (CYP) is a multi-gene superfamily of constitutive and inducible heme-containing monooxygenases, involved in the metabolism of various xenobiotics and endogenous substrates (Blake et al., 2013; Wang et al., 2017; Dutkiewicz and Mikstacka, 2018). The CYP enzyme (P450) is located in the mitochondria and endoplasmic reticulum (Hayran et al., 2020). These enzymes use molecular oxygen and equivalent electrons transferred from the NADPH-P450 reductase in the endoplasmic reticulum or the ferredoxin and ferredoxin reductase in the mitochondria to catalyze the monooxygenase reaction (Renaud et al., 2017). Clopidogrel (CPG) is a classic antiplatelet prodrug widely used to treat thrombosis. Cytochrome P450 (CYP2C19) enzyme mediates the metabolism of CPG to form thiol-containing metabolites (Kazui et al., 2010). The formation of disulfide bonds between the cysteine residues of GSH and the thiol-containing metabolites of CPG to consume intracellular GSH is related to the antiplatelet mechanism of CPG. Inspired by the mechanism of CPG, Wang (Wang et al., 2020) used CPG to consume intracellular GSH in cancer cells expressing CYP2C19 to enhance the efficacy of PDT (Figure 1G). They firstly co-assembled CPG and photosensitizer pyropheophytin (PPa) to form nanoparticles, and then used DSPE-PEG to encapsulate the nanoparticles to form CPG/PPa NPs in order to enhance its stability. CPG and PPa formed stable and nearly spherical nanostructures through strong π-π stacking, hydrophobic interaction, hydrogen bonding and electrostatic interaction. Compared with free PPa, CPG/PPa NPs showed higher cytotoxicity to cancer cells and greater accumulation at the tumor site. After CPG/PPa NPs entering into tumor cell, CPG responded to CYP2C19 to form metabolites containing mercapto alcohols and destroy the redox homeostasis of tumor cells by consuming GSH, showed synergistic anti-tumor effect, and then enhance the efficacy of PDT in the 4T1 breast tumor xenograft model. However, the responsive materials were only modified using PEG. Although PEG modification would enhance the stability and half life of the nanoparticles in the systemic circulation, the negative charge of the PEG might make the modified nanoparticles difficult to enter into the cell. Therefore, the use of ligand molecules such as folic acid, biotin on the surface of nanoparticles might further improve the efficiency of the responsive materials in the PS delivery.

## Summary and Outlook

Enzymes play an important role in physiological and pathological conditions of human body. Enzymes have excellent specificity and selectivity to bind and cleave their substrates, and the reactions are very fast and highly efficient, which provides noval strategy for controlled and targeted drug delivery (Mu et al., 2018). Recent studies have demonstrated that many enzymes are overexpressed in malignant cells and tissues. These overexpressed enzymes provide a unique platform to design ERMs to selectively delivering PSs to tumor tissues and cells. Up to now, series of ERMs, including hyaluronidase-responsive material, matrix metalloproteinase-responsive material, carboxylesterase-responsive material, cathepsin B-responsive material, and cytochrome P450-responsive material, have been researched and developed. Accumulating evidence shows that the ERMs exhibit many fascinating advantages, which not only possess captivating functions of nanomaterials, and also have enzyme-responsive specificity (Mu et al., 2018). These fascinating features make ERMs have promising potential in promoting targeted delivery of PSs and enhancing photodynamic efficacy. In gerenal, enzyme-responsive substrates used in the ERMs are biomolecules such as proteins and peptides. These biomolecules have good biocompatibility and biosafety. However, proteins and peptides easily degaded by exogenous and endogenous enzymes. Thus, the stability of ERMs *in vitro* and *in vivo* should be concerned and improved.

Recently, the physicochemical properties and therapeutic effects of ERMs have been widely studied. The results from the *in-vitro* and *in-vivo* studies have demonstrated that ERMs had a promising prospect in the target delivery of PSs, increase of photodynamic efficacy, and synergetic treatment of photodynamic therapy and chemotherapy/immunotherapy. However, the application of ERMs to deliver PSs in the management of malignacies is still in infancy. Their biosafety, biopharmaceutic and pharmacokinetic performance are rarely been concerned. And few clinical trials were conducted to investigate the precise delivery of PSs for PDT using ERMs. Additionally, tedious preparation, complicated characterization, and uncertainty of the *in-vivo* fate of ERMs are still challenges in the translation of ERMs to clinical applications. Therefore, overcoming the above-mentioned shortcomings and addressing these challenges should be important tasks for translating ERMs as a targeted delivery carrier of PSs for enhancing photodynamic therapy in the clinical settings.
